# Ephrin-A2 regulates excitatory neuron differentiation and interneuron migration in the developing neocortex

**DOI:** 10.1038/s41598-017-12185-x

**Published:** 2017-09-18

**Authors:** Jihane Homman-Ludiye, William C. Kwan, Mitchell J. de Souza, Jennifer Rodger, James A. Bourne

**Affiliations:** 1 Australian Regenerative Medicine Institute, Monash University, Clayton, VIC 3800 Australia; 2 School of Animal Biology, the University of Western Australia, Crawley, WA 6009 Australia

## Abstract

The development of the neocortex requires co-ordination between proliferation and differentiation, as well as the precise orchestration of neuronal migration. Eph/ephrin signaling is crucial in guiding neurons and their projections during embryonic development. In adult ephrin-A2 knockout mice we consistently observed focal patches of disorganized neocortical laminar architecture, ranging in severity from reduced neuronal density to a complete lack of neurons. Loss of ephrin-A2 in the pre-optic area of the diencephalon reduced the migration of neocortex-bound interneurons from this region. Furthermore, ephrin-A2 participates in the creation of excitatory neurons by inhibiting apical progenitor proliferation in the ventricular zone, with the disruption of ephrin-A2 signaling in these cells recapitulating the abnormal neocortex observed in the knockout. The disturbance to the architecture of the neocortex observed following deletion of ephrin-A2 signaling shares many similarities with defects found in the neocortex of children diagnosed with autism spectrum disorder.

## Introduction

Development of the neocortex relies on a balance of progenitor cell proliferation and differentiation in the ventricular zone (VZ) to generate the diversity of neuronal populations. Maintenance of an adequate pool of mitotic progenitors is achieved through signaling pathways promoting symmetrical self-renewing division, while activation of proneural genes promotes the production of neurons through asymmetrical division generating a neuron and a single apical progenitor. During the later stages of corticogenesis, progenitor cells switch to terminal symmetric division producing two neurons, ultimately depleting the progenitor pool^1–3^. The equilibrium between symmetric and asymmetric division is controlled by sets of instructions present in the local environment mediated through cell-cell interactions. The Notch-Delta pathway is particularly important in regulating the transition from one division mode to the other^4^ but guidance molecules, identified for their ability to direct neuronal migration and axon navigation, can also act as fate determinants^5^. Disruption of neurogenesis has dramatic consequences on the establishment of the neocortex, leading to severe malformations and cognitive disorders, including Autism Spectrum Disorder (ASD)^6^.

Glutamatergic excitatory neurons, the largest fraction of neocortical neurons, are born locally in the neocortex, from Pax6+ apical progenitors in the VZ and Tbr2+ basal progenitors in the SVZ^7–9^. Following the upregulation of neuronal markers including NeuN and Tbr1, the newborn excitatory neurons follow a radial migratory route^1,10,11^ to their appropriate laminar position in the developing neocortex.

In contrast, interneurons originate from subcortical domains, combining tangential and radial migration to reach the neocortex^3,12^. In rodents, 90% of neocortical interneurons are born in the ventral telencephalon, including the ganglionic eminences^13^, with the remaining 10% emerging from the diencephalic preoptic area (POA)^14–16^. Interneurons arising from these distinct domains give rise to non-overlapping subtypes occupying specific compartments in the neocortex^17–19^.

Eph/ephrin signaling regulates many aspects of cell interaction in the developing neocortex, including migration and axonal target recognition^20^. Ephrins are membrane bound ligands, tethered to the cell membrane via a GPI link (ephrin-As) or transmembrane region (ephrin-Bs). They interact with Eph tyrosine kinase receptors triggering bi-directional responses: a forward signaling cascade in the receptor-bearing cell and a reverse signaling cascade in the ligand-bearing cell^21–23^. Receptor-ligand interactions principally elicits repulsion, contributing to the migration and segregation of neuronal populations including neocortical excitatory neurons^24,25^ and interneurons^26–28^. Eph/ephrin interactions also regulate other aspects of neocorticogenesis, including brain size through regulation of apoptosis^29^ and progenitor proliferation^30^.

Here we describe focal lamination defects in the neocortex of all adult ephrin-A2 homozygote knockout mice (*efnA2* KO), forming discrete patches of reduced neuronal density. Their distribution is disparate and not restricted to specific neocortical areas, highly reminiscent of similar aberrations observed in the neocortex of children diagnosed with Autism Spectrum Disorder (ASD)^31^. Using *in vivo* and *ex vivo* knockdown approaches we demonstrate that ephrin-A2 contributes to the establishment of excitatory and inhibitory neocortical neurons, with distinct roles in each population. Specifically, ephrin-A2 promotes the migration of interneurons emerging from the POA and modulates the proliferation of apical progenitors in the neocortex. We propose that ephrin-A2 acts as a proneural cell fate determinant, with the loss of ephrin-A2 resulting in abnormal neocortical lamination akin to ASD-associated deficits.

## Results

### Disruption of the neuronal cytoarchitecture in the neocortex of efnA2 KO mice

**Figure 1:**
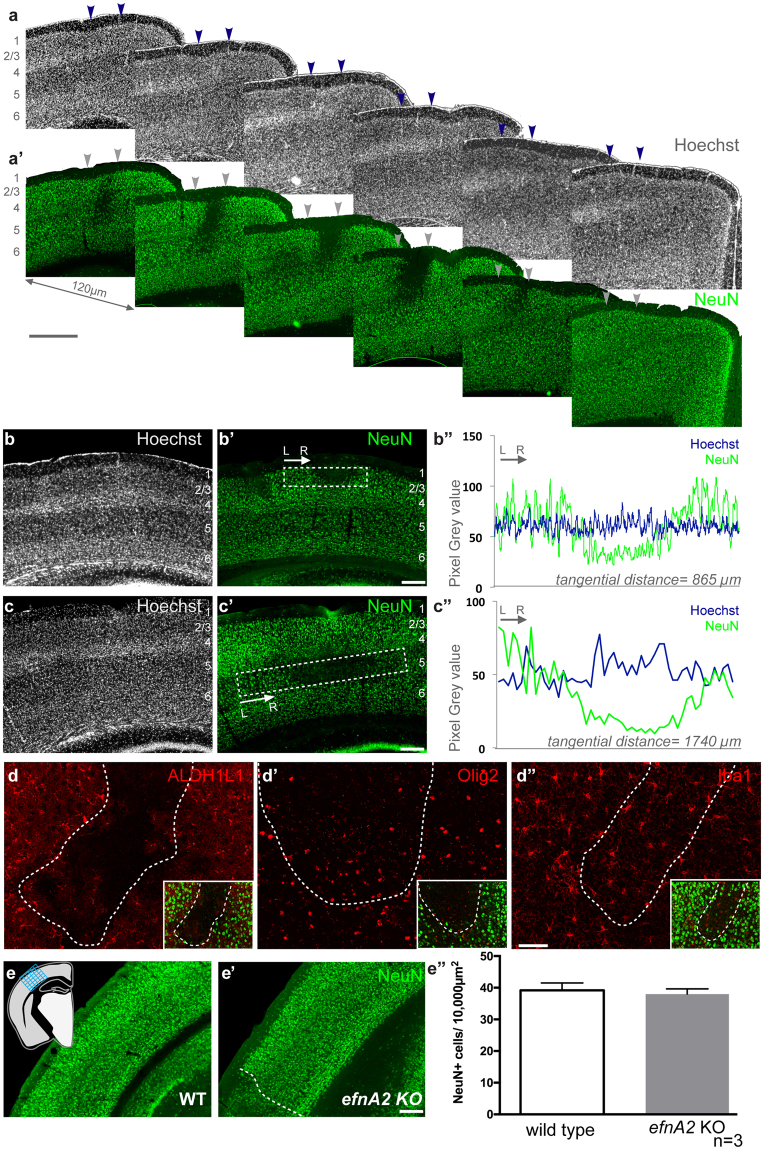
Characterisation of *efnA2* KO adult neocortex. Adjacent coronal sections of *efnA2* KO adult brain stained with the nuclear dye Hoechst (**a**) and labeled with the neuron-specific transcription factor NeuN (**a’**), arrowheads demarcate the maximum width for this patch of low NeuN density. *efnA2* KO neocortex stained with Hoechst (**b**,**c**) and labeled with NeuN (**b’**, **c’**) illustrating abnormal neuronal density in layers 2/3 (**b**, **b’**) and layer 5 (**c**, **c’**). (**b”**, **c”**) Linear graph plot of the signal intensity for each stain measured in the boxed area in (**b** and **c**) respectively. *efnA2* KO neocortex labeled with the astrocytes marker ALDH1L1 (**d**), the oligodendrocyte marker Olig2 (**d’**) and the microglia marker Iba1 (**d”**), hatched lines delineate regions exhibiting abnormal NeuN density ((**d**–**d”**) inset). Quantification of NeuN+ cells density in wild type (**e**) and *efnA2* KO (**e’**) in neocortical regions exhibiting normal laminar architecture ((**e”**) error bars represent SEM; hatched line in (**e’**) illustrates an example of zone excluded from the counting frame) in was quantified across all layers by superimposing a grid over the region of interest (schematic in (**e**)). Scale bars (**a’**) 250 µm; (**b’**,**c’** and **e’**) 100 µm; (**d”**) 50 µm.

The neocortical cytoarchitecture of adult mice lacking *efnA2* appeared normal when labeled with the DNA stain, Hoechst (Fig. 1a). However, labeling with the neuronal marker, NeuN, revealed discrete patches with reduced density of neuronal cell bodies (Fig. 1a’) and dendritic arborisation (Fig. S1a,b’). The patches established sharp boundaries between normal and disrupted zones and extended through the neocortex with the loss of neurons becoming more severe towards the center of the patch, as illustrated on the series of adjacent sections (Fig. 1a’). The phenotype varied in severity, yet occurred with a 100% penetrance, with all mutant brains exhibiting at least one disorganized patch per hemisphere. Their distribution, however, was highly variable and apparently random, with the disruption affecting all layers indiscriminately (Fig. 1b’, layers 2/3; Fig. 1c’, layer 5), confirmed by a signal reduction on the fluorescence intensity profile (Fig. 1b”, c”). The stability of the Hoechst nuclear labeling (Fig. 1b”, c”) and haematoxylin and eosin histology (Fig. S1c, d’) demonstrates that despite the reduction in the density of NeuN+ cells, the neocortical patches were uniformly populated with structurally normal cells. To characterize the identity of the cells localized within the patches we double-labeled *efnA2* KO sections with NeuN to demarcate the abnormal regions, and either ALDH1L1, Olig2, or Iba1 to label astrocytes, oligodendroglia or microglia/macrophages respectively. Both astrocytes and oligodendrocytes were noticeably reduced within the regions lacking neurons, with only a few cells observed close to the border (Fig. 1d–d’), contrasting with the uniform distribution of microglia (Fig. 1d”). The expression profile of 10 additional markers is summarized in Supplementary Table 1. Outside the perturbed regions, the distribution of neurons appeared normal, and the cell density was not significantly different from wild type (number of neurons per 0.01 mm^2^; wild type, 39.2 ± 2.32; *efnA2* KO, 38.01 ± 1.62, n = 3; Fig. 1e–e”).

### Ephrin-A2 modulates the transition of neocortical progenitors towards differentiation

**Figure 2:**
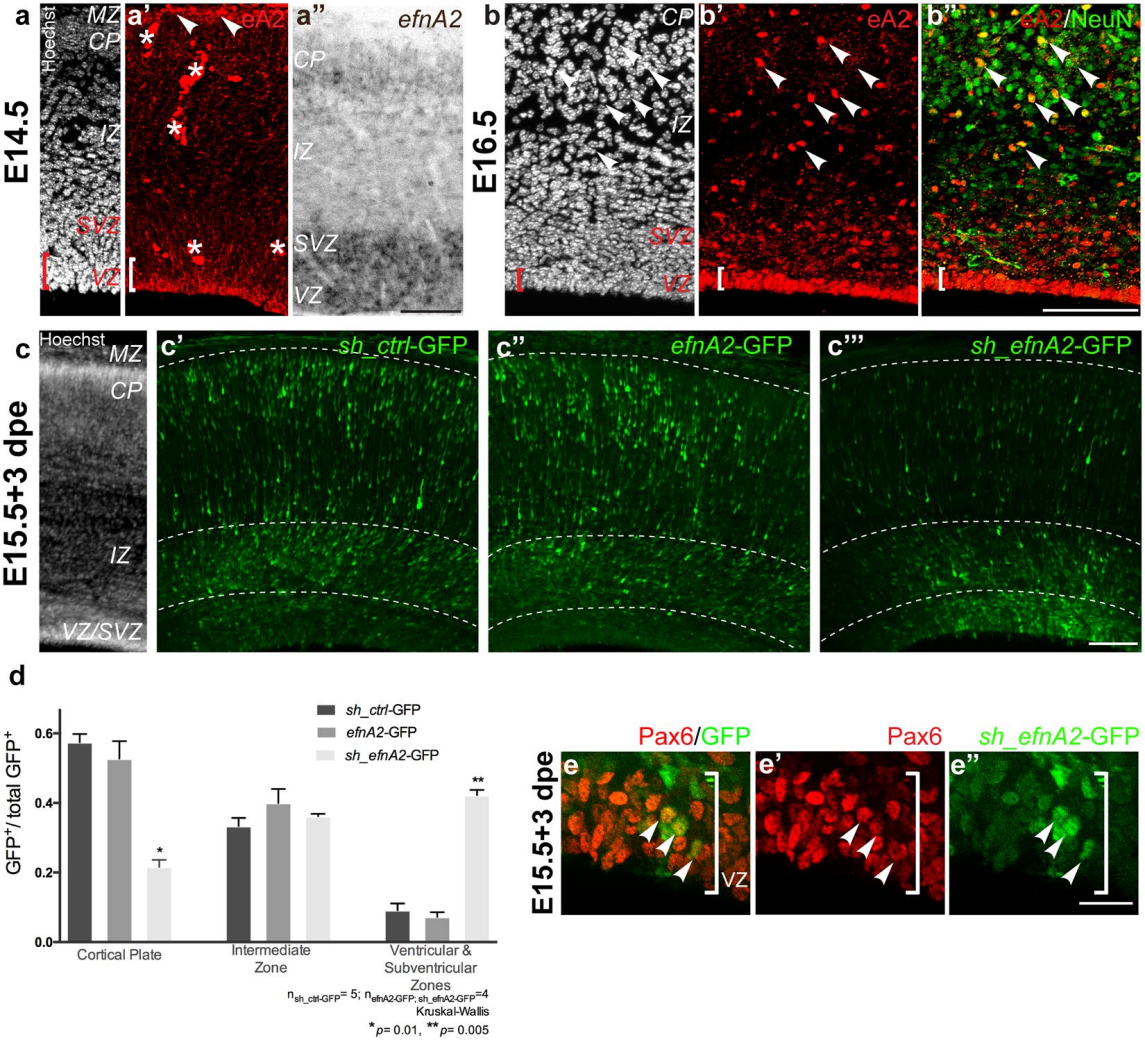
Loss of ephrin-A2 in apical progenitors results in reduced neuronal migration and the accumulation of progenitors in the ventricular zone. (**a**,**b**,**c**) Cytoarchitecture of the developing neocortex demarcated with the nuclear dye Hoechst at E14.5, E16.5 and E18.5 in wild type mice (**a’**) ephrin-A2 immunolabeling at E14.5, (**a”**) *efnA2* messenger *in situ* hybridisation at E14.5 and (**b’**) at E16.5 reveals expression in the cells lining the ventricular surface of the lateral ventricle, in the MZ ((**a’**), arrowheads, *signal autofluorescent blood vessels) and in the cortical plate, double-labeled with NeuN ((**b”**) arrowheads). (**c)** Representative illustration of embryonic neocortex harvested at E18.5 stained with Hoechst following electroporation at E15.5 with (**c’**) GFP-tagged control scrambled shRNA. (**c”**) GFP-tagged construct coding for *efnA2* for overexpression. (**c”’**) GFP-tagged shRNA to knockdown *efnA2* (*sh*_*efnA2*-GFP) (**d**) ratio of GFP+ cells/compartment over the total number of GFP+ cells. (**e**) High magnification of the VZ of *efnA2* knockdown electroporated at E15.5, analyzed at E18.5, *sh_efnA2*-GFP reporter (green), and Pax6 (magenta) (**e’**,**e”**) arrowheads signal electroporated cells also expressing the progenitor cell marker Pax6 (**d**) non-parametric Kruskal-Wallis test, error bar represent SEM *CP* cortical plate, *IZ* intermediate zone, *MZ* marginal zone, *SVZ* subventricular zone, *VZ* ventricular zone Scale bar (**a”**,**b”’**,**c”’**) 100 µm; (**e”**) 20 µm.

The loss of neuronal cell labeling in the neocortex could indicate a developmental role for *efnA2*. We, therefore, examined the developmental expression of ephrin-A2 in the wildtype neocortex. At E14.5 ephrin-A2 was detected in the marginal zone (MZ) and in the VZ, comprised of apical progenitor cells (Fig. 2a–a”). Intense ephrin-A2 expression persisted in the VZ at E16.5, with expression extending to the adjacent SVZ (Fig. 2b’). Ephrin-A2+ cells in the cortical plate (CP) also expressed the neuronal marker NeuN (Fig. 2b”), revealing that ephrin-A2 is present in both progenitor cells and differentiated neurons in the developing neocortex.

To explore the role of ephrin-A2 in the establishment of neuronal populations in the neocortex, we performed loss- and gain-of-function in the apical progenitors lining the ventricular surface of the telencephalon. We electroporated bicistronic plasmid DNA encoding the GFP coding sequence and either ephrin-A2 (*efnA2-GFP*), shRNA against ephrin-A2 (*sh_efnA2-GFP*) or a scrambled shRNA control sequence (*sh_ctrl-GFP*) at E15.5. At 3 days post electroporation (dpe), GFP+ cells were present in all compartments of the embryonic neocortex, with the exception of the acellular MZ (Fig. 2c–c”’) in varying quantities. While the proportion of GFP+ cells transiting through the intermediate zone did not vary between conditions (scrambled control, 0.33 ± 0.02, n = 5; ephrin-A2, 0.40 ± 0.04, n = 4; *sh_efnA2*, 0.36 ± 0.01, n = 4; Fig. 2d), the fraction of cells entering the CP was comparable between control and ephrin-A2 overexpressing conditions but significantly reduced by ephrin-A2 knockdown (scrambled control, 0.57 ± 0.02, n = 5; *ephrin-A2*, 0.53 ± 0.05, n = 4; *sh_efnA2* 0.22 ± 0.02, n = 4; *p* = 0.01, Kruskal-Wallis test; Fig. 2d). Only a small fraction of control and ephrin-A2 overexpressing cells remained in the neurogenic ventricular and subventricular layers and this proportion was significantly increased following ephrin-A2 knockdown (scrambled control, 0.09 ± 0.02, n = 5; ephrin-A2, 0.07 ± 0.01, n = 4; *sh*_*efnA2* 0.42 ± 0.01, n = 4; *p* = 0.005, Kruskal-Wallis test; Fig. 2d).

Failure of cells to exit the neurogenic compartment following ephrin-A2 knockdown suggests that ephrin-A2 signaling is necessary for newly generated neurons to migrate out of the neurogenic compartment. Alternatively, ephrin-A2 may act at an earlier stage of neurogenesis, with the reduction in ephrin-A2 resulting in progenitors failing to commit to a differentiated neuronal fate and arrest in the VZ.

To determine the identity of the *sh_efnA2*-GFP cells, we tested for the expression of progenitor markers and demonstrated that *efnA2* knockdown cells persisting in the neurogenic zone maintain the expression of Pax6 (Fig. 2e–e”). In the embryonic neocortex, Pax6 expression is restricted to apical progenitors dividing at the ventricular surface^9^. Pax6 controls neocortical cell numbers by regulating the balance between proliferation and cell cycle exit^32^. Altogether, our results suggest that loss of ephrin-A2 causes progenitors to remain in the VZ and maintain Pax6 expression, which ultimately would result in increased proliferation at the expense of neuronal differentiation.

### Ephrin-A2 reverse signaling regulates neocortical progenitor proliferation

**Figure 3:**
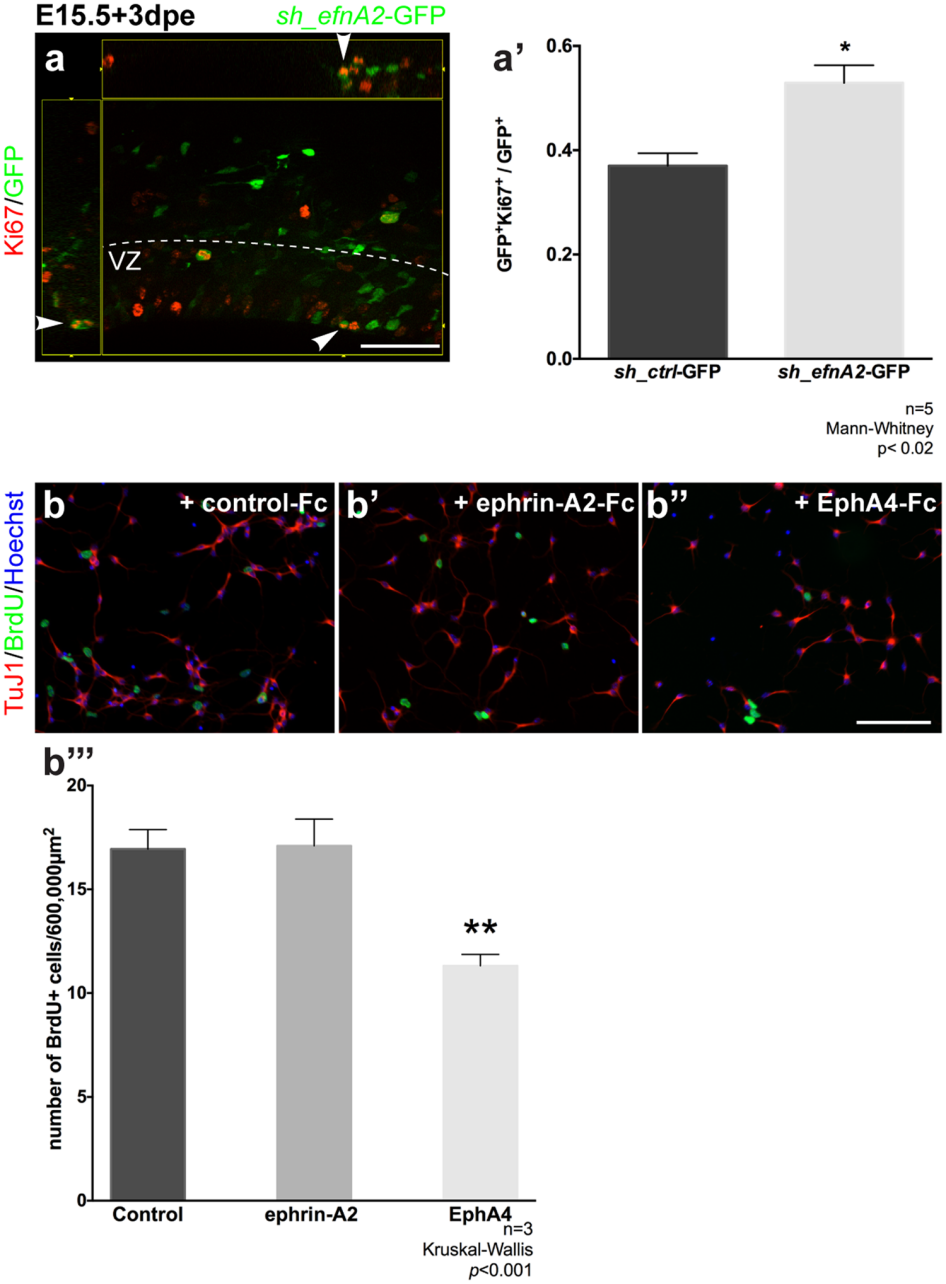
ephrin-A2 reverse signalling inhibits cortical progenitor proliferation. (**a**) Embryonic neocortex electroporated at E15.5 with GFP-tagged control scrambled shRNA (*sh*_*ctrl*-GFP) and GFP-tagged shRNA to knockdown *efnA2* (*sh*_*efnA2*-GFP) and analyzed 3 days post electroporation (dpe) were labeled with the proliferation marker Ki67 (**a’**) quantification of the mitotic fraction of electroporated GFP+ cells in the ventricular zone (n = 5; *p* = 0.01; Mann-Whitney test, error bars represent SEM). (**b**) Dissociated E14.5 neocortex cultured in presence of BrdU and clustered Fc fragment (control), (**b’**) ephrin-A2-Fc and (**b”**) EphA4-Fc, labeled with the neuronal marker TuJ1 (magenta), the thymidine analogue BrdU (green) and counterstained with the nuclear dye Hoechst (white) (**b”’**) the number of BrdU+ cells was quantified to assess the proliferation after 24 hours in culture. (n = 3; *p* < 0.001; Kruskal-Wallis test, error bars represent SEM). Scale bar (**a**) 50 µm; (**b”**) 100 µm.

We further investigated the relationship between ephrin-A2 and cell cycle and whether this effect was mediated through forward or reverse signalling using a combination of *in utero* electroporation and *in vitro* approaches. To determine if ephrin-A2 knockdown at E15.5 affected the proliferation of apical progenitors, we quantified the proportion of proliferative cells within the GFP+ population in the VZ at 3dpe (Fig. 3a). The fraction of GFP+ cells expressing the proliferation marker Ki67 increased in *sh_efnA2-GFP* electroporated mice compared to controls (*sh_ctrl-*GFP, 0.37 ± 0.05; sh_*efnA2*, 0.53 ± 0.08, n = 5, *p* = 0.01, Mann-Whitney test; Fig. 3a’). This result confirms that reducing ephrin-A2 causes apical progenitors to remain in a proliferative undifferentiated progenitor state.

It is common for cells expressing ephrin ligands to co-express Eph receptors, segregated in separate microdomains to prevent cis-activation^33^. Therefore, the phenotype observed following loss of ephrin-A2 could result from a deficit in forward or reverse signaling. To discriminate between forward or reverse signaling in the control of apical progenitor differentiation, we dissociated the neocortices of E14.5 wild-type mice and plated the cells at low density to limit cell-cell interaction. Next, we added clustered recombinant frame chain (Fc) ephrin-A2 (ephrin-A2-Fc) to induce ephrin-A2 forward signaling (Fig. 3b’); reverse signaling by adding clustered EphA4-Fc (Fig. 3b”); with clustered Fc fragment used as a negative control (Fig. 3b). While clustered EphA4-Fc has been demonstrated to activate ephrin-A2 reverse signaling^28^, it could potentially bind ephrin-A5 as well. However, the absence of ephrin-A5 expression in the embryonic neocortex between E13.5 and E16.5^34^ ensured that ephrin-A2 reverse signaling was selectively activated in our culture system. BrdU was used to tag cells in the S-phase of the cell cycle. We found that the number of BrdU+ cells did not vary between control and ephrin-A2-Fc conditions. However, activation of ephrin-A2 reverse signaling significantly reduced the number of BrdU+ cells (control-Fc, 16.9 ± 0.94; ephrin-A2-Fc, 17.1 ± 1.3; EphA4-Fc, 11.3 ± 0.55, n = 3, *p* < 0.001, Kruskal-Wallis test; Fig. 3b”’). Therefore, the increase in proliferation of neocortical progenitors following ephrin-A2 knockdown is likely to be mediated via a reduction in ephrin-A2 reverse signaling rather than a reduction in forward signaling on Eph receptor bearing cells.

### Knockdown of ephrin-A2 in neocortical progenitors reduces the production of excitatory neurons

**Figure 4:**
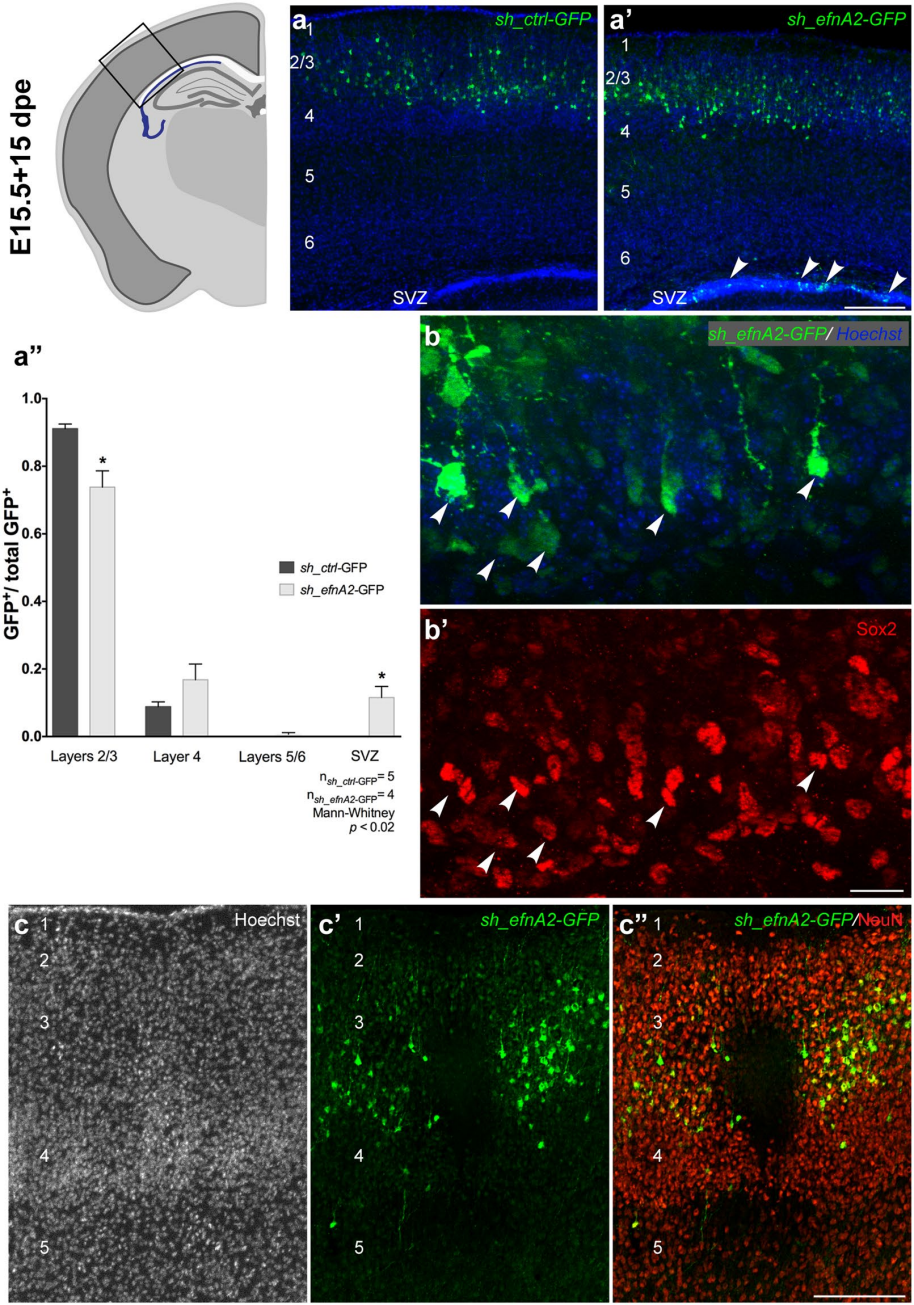
Loss of ephrin-A2 prevents neuronal differentiation and disrupts the distribution of excitatory neurons in the postnatal neocortex. Laminar distribution of GFP+ cells at P12 following electroporation at E15.5 (15 days post electroporation *dpe*) with (**a**) GFP-tagged control scrambled shRNA (*sh*_*ctrl*-GFP) and (**a’**) GFP-tagged shRNA to knockdown *efnA2* (*sh*_*efnA2*-GFP), arrowheads in (**a’**) signal GFP + cells in the subventricular zone (SVZ) (**a”**) the number of GFP+ cells was quantified in each compartment and expressed as a ratio of the total GFP population (**a”**) scrambled control shRNA, n = 5; *efnA2* shRNA, n = 4; *p* < 0.02; Mann-Whitney test, error bars represent SEM). GFP+ cells knockdown for *efnA2* remaining in the SVZ ((**b**) arrowheads) express the stem cell marker Sox2 ((**b’**) arrowheads). (**c**) Loss of *efnA2* does not affect the laminar architecture as revealed by Hoechst staining although distribution of the GFP+ electroporated cells (**c’**) and density of NeuN+ cells (**c”**) was profoundly altered. Scale bar (**a’**,**c”**) 200 µm; (**b’**) 20 µm.

To investigate the long-term effect of ephrin-A2 knockdown on cortical progenitors, animals electroporated with *sh_efnA2*-GFP at E15.5 were analyzed at P12. The laminar distribution of electroporated neurons was similar between control and ephrin-A2 knockdown animals, with the majority of GFP+ cells present in the supergranular layers (Fig. 4a,a’). However, some GFP+ cells remained in the SVZ of ephrin-A2 knockdown mice (Fig. 4a’, arrowheads) whereas there were very few GFP+ cells in the SVZ of *sh_ctrl-GFP* mice. Consequently, the proportion of *sh_efnA2*-GFP+ cells in the supergranular layers was reduced compared to the control (layer 2/3: scrambled, 0.91 ± 0.0.3, n = 5; *sh-efnA2*, 0.74 ± 0.09, n = 4, p = 0.016, Mann-Whitney test; Fig. 4a”), and the proportion of *sh_efnA2*-GFP+ cells in the SVZ was increased relative to controls (scrambled, 5.7 × 10^−5^ ± 1.3 × 10^−4^, n = 5; *sh-efnA2* 0.115 ± 0.003, n = 4, p = 0.018, Mann-Whitney test; Fig. 4a”). We confirmed that the GFP + cells in the SVZ of *sh_efnA2*-GFP mice expressed the transcription factor Sox2, consistent with a progenitor identity (Fig. 4b–b’, arrowheads). Therefore, while the vast majority of cells electroporated with the control shRNA differentiated into neurons and migrated to supergranular layers, over 10% of ephrin-A2 knockdown cells maintained their progenitor identity for 2 weeks after electroporation.

Despite cellular density appearing normal in the neocortices of ephrin-A2 knockdown animals (Fig. 4c), we detected discontinuities in the laminar distribution of GFP+ neurons in the electroporated hemispheres (Fig. 4c’). The supergranular layers of ephrin-A2 knockdown neocortices exhibited discrete regions devoid of GFP + cells which were also devoid of NeuN + cells (Fig. 4c”), similar to the cortical defects (patches) we described in the neocortex of adult *efnA2* KO (Fig. 1). Therefore, loss of ephrin-A2 in apical progenitors through targeted knockdown is sufficient to phenocopy the abnormal laminar architecture observed in the knockout animals.

### Reduction of the calbindin-positive interneuron fraction in the supragranular layers of efnA2 KO mice

**Figure 5:**
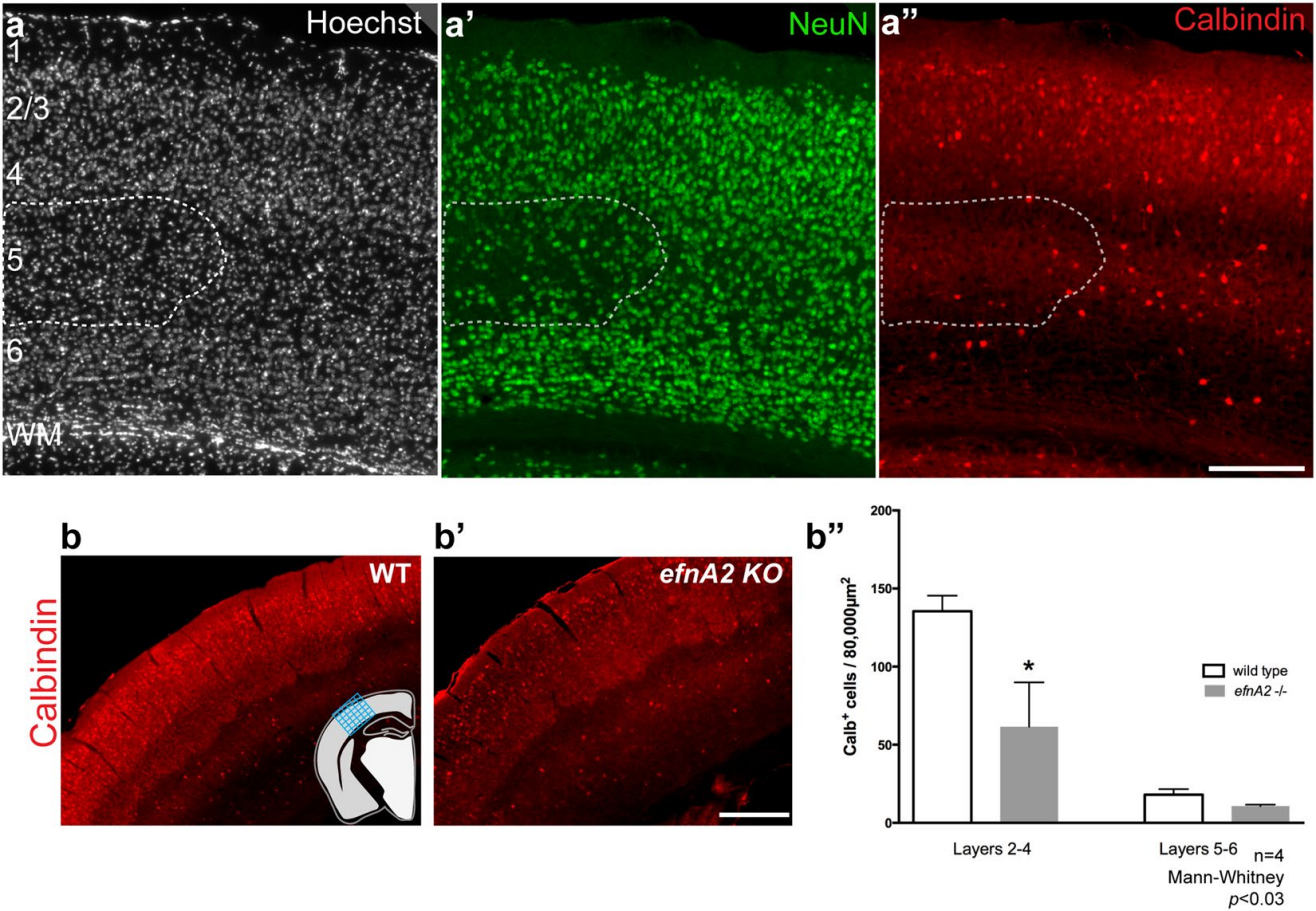
Dramatic reduction of calbindin expression throughout the neocortex of *efnA2* KO animals. (**a**) Hoechst nuclei staining of *efnA2* KO neocortex demarcating the laminar architecture of the neocortex, double-labeled with (**a’**) the neuron-specific transcription factor NeuN, (**a”**) with the interneuron marker calbindin (Cb). Hatched boxes in (**a**–**a”**) delineate the patches of lower neuronal density. (**b**–**b’**) Example of Cb staining in the neocortex of adult wild type and *efnA2* KO animals, schematic in (**b**) illustrates the region that was analyzed along the antero-posterior axis of the brain by applying a grid over the zone counted, regions showing abnormal density were demarcated by NeuN double-labeling and excluded. (**b”**) density of Cb+ interneurons across the neocortical layers of WT and *efnA2* KO (n = 4, p < 0.03, Mann-Whitney test, error bars represent SEM); *WM* white matter; scale bar (**a”** and **b’**) 200 µm.

To determine if loss of ephrin-A2 also affected interneurons, we compared calbindin immunolabeling between *efnA2* KO and wild type control. We identified a severe reduction in interneurons density within the patches of reduced NeuN+ labeling in *efnA2* KO (Fig. 5a–a”, hatched region). Calbindin + interneurons were present in the rest of the neocortex, exhibiting a distribution profile comparable to the wild type control with the majority of the cells populating the supragranular layers 2/3 and fewer cells in the infragranular layers 5/6 (Fig. 5b–b’). However, the density of calbindin+ cells throughout the entire neocortex, excluding the abnormal patches, was significantly reduced (number of cells in layers 2 to 4 per 80,000 µm^2^; wild type, 135.50 ± 4.96; *efnA2* KO, 61.59 ± 14.17, *p* = 0.029, n = 4, Mann-Whitney test; Fig. 5b”). This suggests that ephrin-A2 plays a major role in the establishment of this fraction of inhibitory neurons but, unlike the focal effect observed in the case of excitatory neurons, loss of ephrin-A2 affects interneurons throughout the entire neocortex suggesting that the ligand has distinct roles in excitatory and inhibitory neurons development.

### Ephrin-A2 is expressed by superficially migrating interneurons

**Figure 6:**
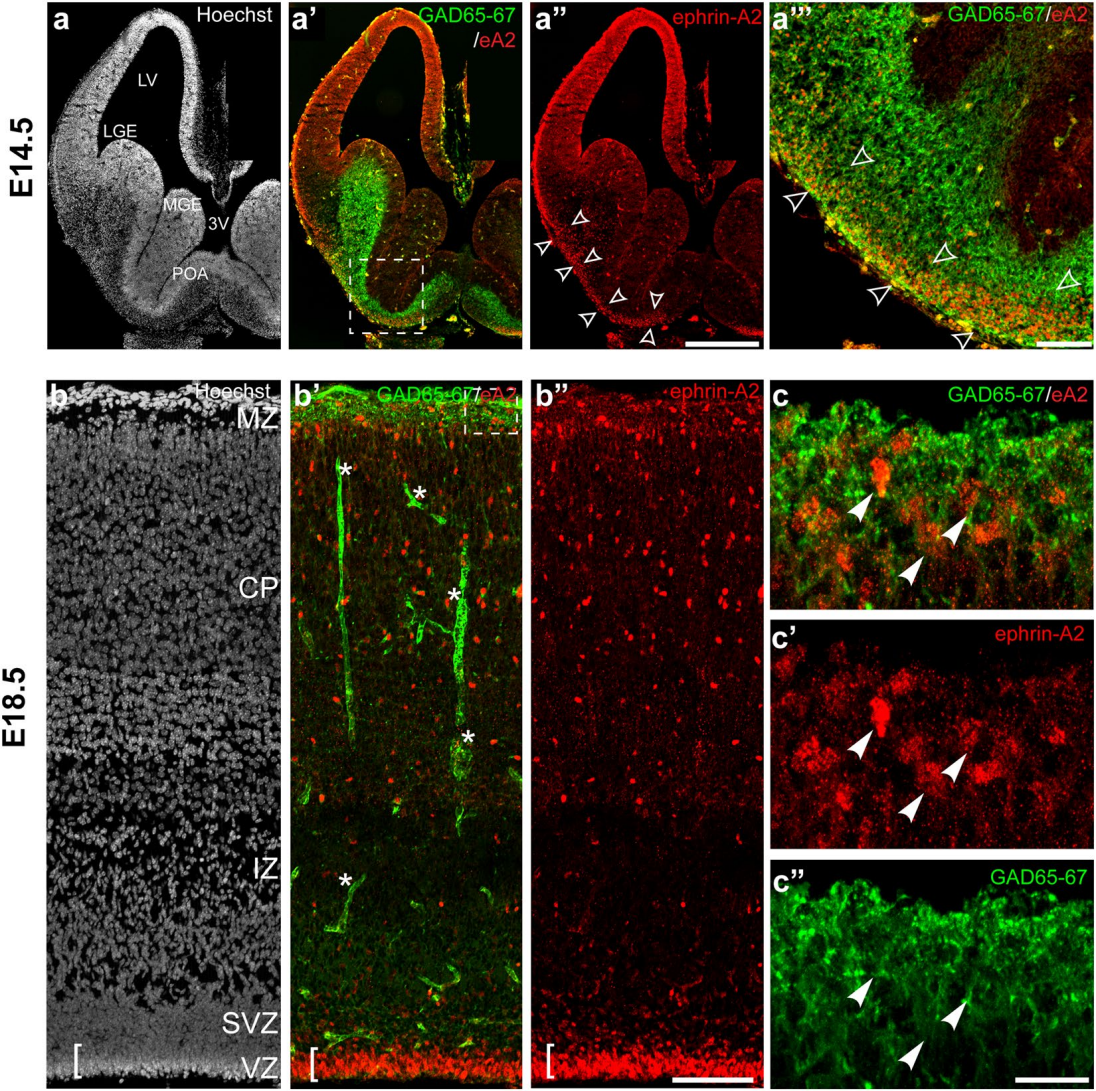
ephrin-A2 is expressed in migrating interneurons. (**a**) At E14.5 LGE, MGE and POA neurogenic zones lining the ventricular surface have high cell density and appear brighter with Hoechst staining (**a’**) Cells expressing ephrin-A2 colocalize with the interneuron marker GAD65–67 (boxed region magnified in (**a”’**)) (**a”**) ephrin-A2-positive cells form a stream stretching along the ventral surface of the brain from the POA to the neocortex (open arrowheads). (**b**) Hoechst staining reveals the lamination of the neocortex at E18.5 (**b’**) Interneurons expressing GAD65–67 populating the neocortex through the MZ colocalize with ephrin-A2+ cells are located in the VZ, the CP and the MZ (*signal blood vessels). (**b”**) ephrin-A2 expressing cells are also present in the CP and the VZ (**c**) High magnification of the boxed region in (**b’**) highlighting ephrin-A2+/GAD65–67+ interneurons (arrowheads) in the MZ entering the CP (**c’**) ephrin-A2 expression (**c”**) GAD65–67 expression reveals the radial orientation of the cell processes as interneurons enter the CP *CP* cortical plate, *IZ* intermediate zone, *LGE* lateral ganglionic eminence, *LV* lateral ventricle, *3 V* third ventricle, *MGE* medial ganglionic eminence, *MZ* marginal zone, *POA* preoptic area, *SVZ* subventricular zone, *VZ* ventricular zone. Scale bar (**a”**) 500 µm, (**b”**) 100 µm, (**c”**) 20 µm.

At E14.5, interneurons emerging from two transient telencephalic regions, the medial ganglionic eminence (MGE) and the POA (Fig. 6a) and characterized by the expression of GAD 65 & 67 also express ephrin-A2 (Fig. 6a’; boxed region magnified in 6a”’). Ephrin-A2+ cells were organized in a continuous stream extending from the POA to dorsolateral telencephalon, (Fig. 6a”; open arrowheads), correlating with the superficial migratory stream. Interneurons migrating along the superficial migratory stream reach the neocortex at the level of the MZ as illustrated by the dense GAD65 & 67 staining observed in the MZ at E18.5 (Fig. 6b’). Double-labeling revealed the presence of ephrin-A2+ cells in the MZ at this stage, in addition to the dense population in the VZ and scattered throughout the CP observed at earlier stages (Fig. 6b”). Ephrin-A2 expressing cells in the MZ co-express GAD 65 & 67 (Fig. 6c–c”) confirming their interneuronal identity. Altogether, this expression profile demonstrates that interneurons originating from the MGE and the POA upregulate ephrin-A2 expression after exiting the MGE and POA neurogenic zones, characterized by the high nuclei density (Fig. 6a) and the lack of GAD 65 & 67 expression (Fig. 6a’), as they join the superficial migratory stream to reach the neocortex. They maintain the ligand expression after completing the tangential phase of their migration to the MZ and dispersing radially in the CP. The lack of ephrin-A2 expression in the VZ of the 3rd ventricle suggests that the ligand does not participate in progenitor proliferation but might play a role in regulating tangential migration.

### Ephrin-A2 promotes the migration of interneurons emerging from the POA

**Figure 7:**
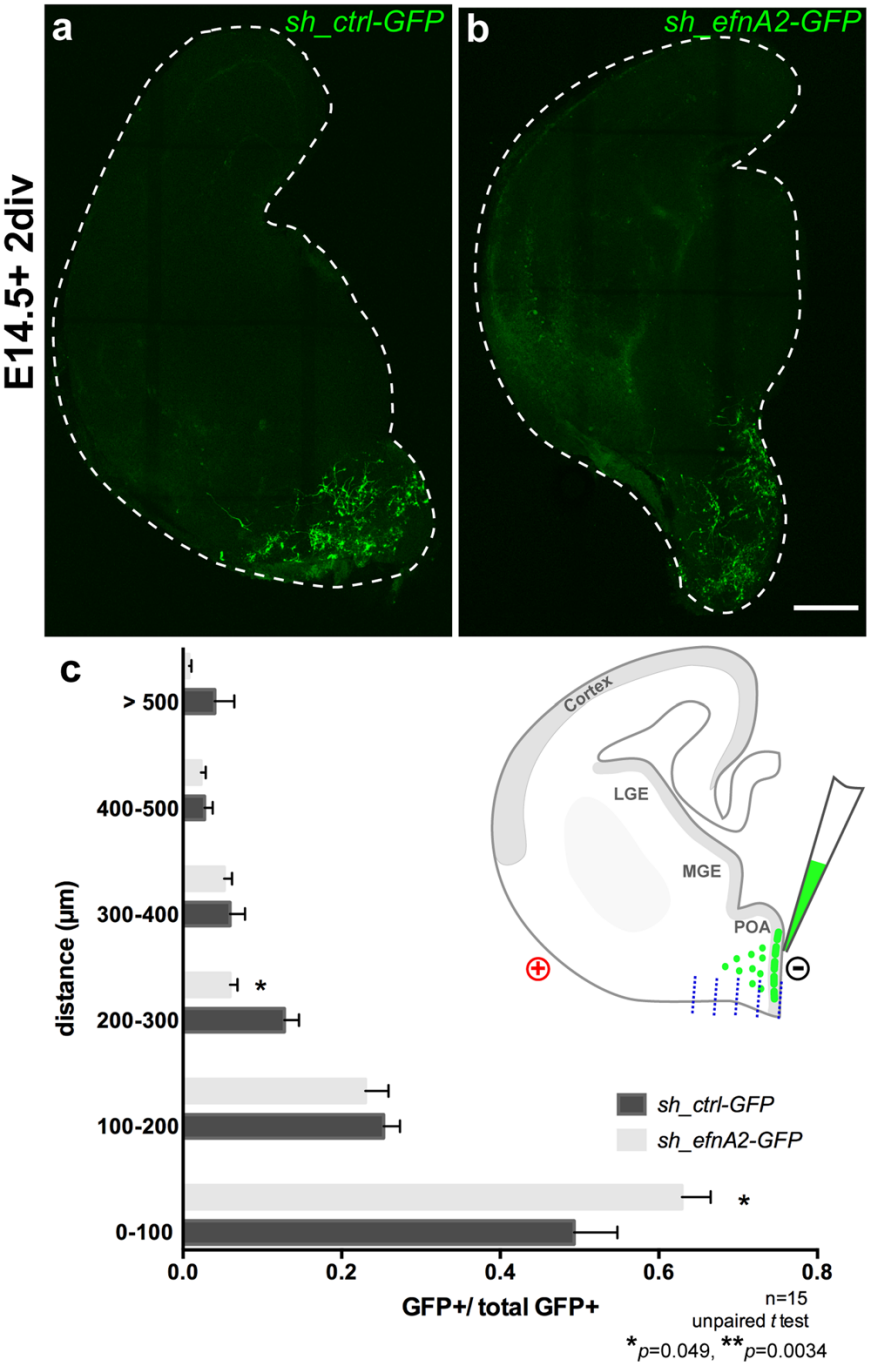
Loss of ephrin-A2 reduces migration of POA-born interneurons. Coronal slices of brain electroporated at E14.5 with (**a**) GFP-tagged control scrambled shRNA (*sh*_*ctrl*-GFP) or (**b**) GFP-tagged shRNA to knockdown *efnA2* (*sh*_*efnA2*-GFP) and cultured for 48 hours (2 days *in vitro div*) (**c**) Electroporated cells migration was established by calculating the ratio of GFP+ cells in 100 µm bin (origin = electroporation site) over the total number of GFP+ cells in the section (schematic in (**c**) *see blue dashed lines*) revealing a larger fraction of cells closer to the site of electroporation following *sh*_*efnA2*-GFP electroporation (n = 15 independent sections from separate embryos analyzed for each condition, p = 0.049; 0.0034, unpaired *t* test, error bars represent SEM) Scale bar (**b**) 200 µm.

Interneurons emerging from the POA follow a superficial migratory stream along the ventral surface of the brain and are kept segregated from their MGE-born counterparts through EphA4-ephrinB3 mediated bidirectional repulsion^27^. To elucidate the function of ephrin-A2 in regulating the migration of POA-born interneurons we undertook an *ex vivo* electroporation approach, enabling us to specifically knockdown ephrin-A2 in the POA (Fig. 7a,b). After 2 days of *in vitro* culture (2div), we counted the number of GFP+ cells in 100µm-wide bins parallel to the medial edge of the VZ to quantify the extent of cell migration from the site of tissue electroporation (*see schematic* Fig. 7). Fewer than 50% of cells electroporated with control DNA remained within 100 µm of the medial edge of the VZ of the POA whereas more than 60% of cells electroporated with ephrin-A2 knockdown shRNA remained within this region (scrambled, 0.493 ± 0.054; *sh*-*efnA2* 0.629 ± 0.036, n = 15 sections/condition, p = 0.049, Student’s t-test; Fig. 7c). The reduced migration of *sh_efnA2* electroporated cells was also reflected by a significant decrease in the proportion of cells that had migrated from 200–300 µm (scrambled, 0.128 ± 0.018; sh-*efnA2* 0.059 ± 0.01, n = 15, p = 0.0034, Student t-test; Fig. 7c). Overall, our data suggest that knockdown of ephrin-A2 within interneurons emerging form the POA reduces their rate of tangential migration, rather than completely inhibiting their ability to reach the neocortex.

## Discussion

Our study demonstrates that loss of ephrin-A2 during development leads to perturbations in the establishment of neocortical layers, resulting in the formation of discrete, random, patches depleted of neurons. Although Eph/ephrins are traditionally known for regulating migratory events, our results suggest that alteration of the neocortical architecture in the absence of *efnA2* results from the abnormal production of neurons before migration to the cortical plate is initiated. Although the lamination defects in *efnA2* KO neocortex were spatially restricted to patches, with the neuronal density comparable to control animals in unaffected regions, we uncovered a significant loss of calbindin-expressing neocortical interneurons in apparently ‘normal’ regions of the neocortex, suggesting that in addition to differentiation of pyramidal neurons, ephrin-A2 also contributes to the establishment of neocortical interneurons.

Comparable local disruptions of the neocortical layers observed in *efnA2*; *A*3; *A5* triple knockout mice^24^ have been attributed to the abnormal lateral dispersion of excitatory neurons in the cortical plate, without specifying the role of each gene. The presence of ephrin-A2+ neurons in the cortical plate from E14.5 to E18.5, supports the hypothesis that the ligand participates in the regulation of neuronal migration. However, during these stages, ephrin-A2 is predominantly expressed in the VZ suggesting that it has additional functions. The VZ is comprised of Pax6+ apical progenitors^9^ undergoing mainly asymmetric division (78%), to generate a self-renewed apical progenitor and either a neuron (65.8%) or a basal progenitor. Only 10% of divisions are symmetrical with both daughter cells remaining in the VZ^2,35^. Accumulation of proliferative Pax6+ cells in the VZ following reduction of *efnA2* suggests that a larger proportion of apical progenitors divided according to a symmetrical self-renewing mode and re-entered the cell cycle instead of generating excitatory neurons.

**Figure 8:**
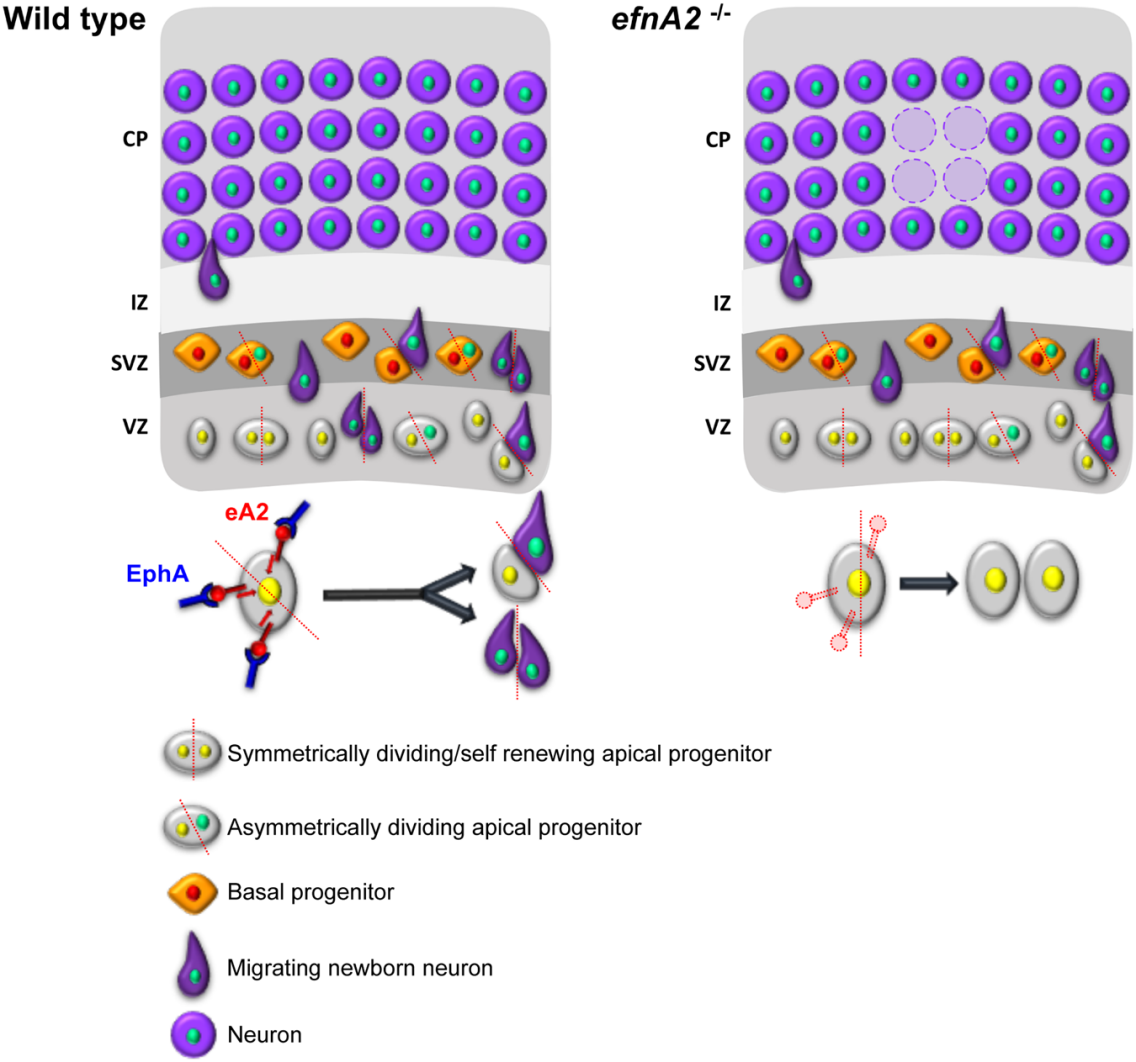
Proposed model for ephrin-A2’s role. In normal wildtype, activation of ephrin-A2 reverse signalling following interaction with Eph promotes the asymmetrical or terminal symmetrical division of apical progenitors in the VZ to generate neurons through activation of a pro-neural pathway. In knockout animals, in absence of pro-neural signals, apical progenitors undergo symmetrical self-renewing division leading to a deficit of glutamatergic neurons in the developing cortical plate and accumulation of progenitors. These missing neurons are apparently replaced by other cells which identity is yet to be determined.

Consistent with these findings, fewer neurons deriving from progenitors in the VZ were present in the postnatal neocortical layers, with dramatic disruption of the laminar architecture observed in extreme cases, replicating the *efnA2* KO phenotype. Our results reveal that ephrin-A2 acts as an early cell-fate determinant in apical progenitors, directing their progeny towards a differentiated neuron identity and repressing cell division. The lack of ephrin-A2 signaling promotes cell proliferation and maintains the apical progenitor pool leading to abnormal neuron production and alteration of the neocortical architecture (see Fig. 8).

The signaling elicited by the interaction of ephrin-A2 with an Eph receptor is bidirectional, activating transduction pathways in both ligand and receptor-bearing cells. Although activation of EphA4 forward signaling by intrinsic ephrin-B1^30^ or extrinsic ephrin-A5^34^ promotes apical progenitor proliferation and neuron production in the mouse embryonic neocortex, the addition of clustered ephrin-A2 to dissociated embryonic neocortex cultures had no effect on cell proliferation. Consistent with evidence that ephrin-A2 reverse signaling inhibits the proliferation of adult stem cells in the mouse SVZ^36^, we observed reduced proliferation following activation of ephrin-A2, demonstrating that ephrin-A2 function on apical progenitors is mediated through reverse signaling. Ephrin-A2 is tethered to the cell membrane via a GPI anchor, preventing it from directly activating intracellular transduction molecules. Instead, activated ephrin-A2 molecules form complexes with other transmembrane molecules and “highjack” their signaling pathway^20^. The neurotrophin receptors p75 and TrkB, critical for neural progenitors commitment to a neuronal fate by inhibiting cell division and promoting neuronal maturation^37,38^, are privileged co-receptors for ephrin-A2^39,40^.

While ephrin-A2 reverse signaling is activated by EphA7 in the adult SVZ^36^, we were not able to identify a unique receptor responsible for eliciting this pathway in the embryonic neocortex with EphA3, A4, A5 and A7 all being strongly expressed in the neocortex. EphA3 is an interesting binding partner candidate for ephrin-A2 expressed on apical progenitors in the neocortical VZ. It has previously been demonstrated that ephrin-A2/EphA3 interaction elicits the recruitment of the metalloproteinase ADAM10 resulting in cleavage of ephrin-A2 from the cell surface^41,42^. This molecular interaction could also underlie the observed scarcity of ephrin-A2 beyond the VZ in the mouse neocortex.

The significant reduction of calbindin labeling in *efnA2* KO neocortex suggests that ephrin-A2 participates in the establishment of neocortical interneurons. Unlike our observations in the neocortex, ephrin-A2 is not expressed in the germinal layers of the regions where interneurons are born, principally the MGE^14,15^ and the POA. Ephrin-A2 is detected in the superficially migrating interneurons, in agreement with recent demonstration that it promotes the motility of MGE-derived interneurons^28^. Our new evidence confirms that this role is conserved in POA-born interneurons. However, cell migration is not arrested in absence of ephrin-A2; it is only delayed with interneurons eventually reaching the neocortex, albeit later than normal. Therefore, the reduction in neocortical calbindin labeling is unlikely to correspond to an absence of interneurons due to abnormal migration.

Although the calbindin + population is reduced by 50% across the supergranular layers, the overall neuronal density is normal in *efnA2* KO neocortex, beside the patches. Therefore, the decrease in calbindin + cells might not reflect an actual absence of supergranular interneurons but instead abnormal expression of the marker itself. As a member of the calcium-binding molecule family, Calbindin becomes upregulated in mature interneurons to buffer Ca^2+^ flux^43^. Interneuron maturation is dependent upon correct synaptogenesis and pruning, which are both under the control of Eph/ephrin signaling^44,45^. In particular, ephrin-A2 reverse signaling utilizes the TrkB transduction pathway^40^ known to modulates calbindin upregulation in maturing interneurons^46,47^. Similarly to *efnA2* KO, *TrkB* deficient animals exhibit a reduction of calbindin + interneurons, consequence of the downregulation of the cell marker and not an actual loss of interneurons. In addition, delayed migration following loss of ephrin-A2 implies that interneurons are exposed to an aberrant, more mature, environment when they disperse through the neocortex. Therefore, the abnormal signals they are exposed to and the atypical network of connections they attempt to integrate are likely to affect the maturation of interneurons lacking ephrin-A2, ultimately affecting calbindin expression.

Thus, the dramatic interneuronal phenotype we report in *efnA2* KO results from an impaired execution of maturation programs leading to lower than normal calbindin expression and not an actual depletion of neocortical interneurons.

*efnA2* KO mice exhibit an interesting behavioral phenotype characterized by perturbed social interactions and repetitive grooming which reflects aspects of autistic-like behaviors in humans^48^. Members of the Eph/ephrin family, including EphA6 and EphB2, have been associated with Autism Spectrum Disorder (ASD) in recent genome-wide association studies (GWAS)^49,50^ but no studies have yet linked ephrin-A2 with the disorder. It could be a consequence of technical limitations as sequences with high GC content (ephrin-A2 GC ratio = 67%) are poorly captured in exome sequencing studies^51^. The discrete and indiscriminate disruption of neuronal density we observed in *efnA2* KO neocortex has, however, been reported in at least one systematic anatomical study of the neocortical architecture of children diagnosed with ASD^31^. The lamination defects they report in all ASD samples and absent from age-matched controls share many of the characteristics we observe in *efnA2* KO mice, including the spatially restricted configuration with sharp borders and the stochastic distribution pattern. While the high degree of variability regarding neocortical areas and layers disrupted in ASD patients could be anticipated considering the large spectrum of symptoms observed in patients and the long list of genes associated with ASD, it is less expected in an animal model bearing a mutation in a single gene.

The phenotypic consistency observed between ASD samples and the *efnA2* KO mice suggests a strong link between Eph/ephrin and ASD. Here, we demonstrate a novel mechanism by which ephrin-A2 regulates neurogenesis and determine that disruption of ephrin-A2 activity in the VZ is a potential contributor to ASD. Furthermore, our findings establish a new time-frame for the cellular mechanisms underpinning ASD, suggesting that they can occur as early as the initial stages of neocorticogenesis.

## Materials and Methods

### Mice

All experiments and procedures were conducted in accordance with the Australian Code of Practice for the Care and Use of Animals for Scientific Purposes and were approved by Monash University and the University of Western Australia Animal Ethics Committees. Animals were housed in cages of 2–5 in a controlled environment with a 12 hour light/dark cycle. Pregnant mice were housed individually 48 h prior to surgery to allow habituation to their environment. Adult *efnA2* homozygous knockout (background strain C57bl/6j) brains were obtained from the University of Western Australia. For *in utero* electroporation and tissue collection, time-mated 8 weeks old pregnant C57bl/6j mice were obtained from local colonies. The plug date was defined as E0.5, and the day of birth was defined as P0. A minimum sample size of n = 3 was used.

### In utero electroporation

The *enfA2* knockdown construct tagged with GFP was a gift from Professor Jürgen Bolz (*see* ^28^ for details). A GFP-tagged scrambled shRNA was used as a control. Mouse ephrin-A2 was amplified using the forward GGGCGGCAGATCTCCAAG and reverse AAAAGTCCACCCCACTCCC primers and the resulting 1564 bp fragment cloned into pCAGS-IRES-GFP. Pregnant mice were assigned a construct using a simple randomization method. Time-mated pregnant females were anesthetized with isoflurane (4% for induction and 2% to maintain) on a heated mat. The abdominal cavity was open and the uterine horns exposed. The plasmid suspension (0.5 µg.µL^−1^ in PBS) was injected in the lateral ventricle using a glass needle and an electric current (35 V, 50 ms, 950 ms interval ×5 pulses) was delivered using 1 mm platinum tweezertrodes (BTX) connected to EMX BTX (Harvard apparatus). Embryos were harvested 3 or 15 days post electroporation (dpe).

### *Ex vivo* electroporation and slice assay

E14.5 embryonic brains were dissected in Complete HBSS medium (*see* ^52^, the two hemispheres separated and the randomly assigned plasmid solution injected directly in the POA followed by application of an electric current (25 V, 50 ms, 250 ms interval ×4 pulses) using 1 mm platinum tweezertrodes (BTX). Electroporated hemispheres were embedded in 4% low-melting point agarose (LMP, Sigma) and sectioned using a vibratome into 300 µm thick coronal slices which were transferred on the membrane of a cell culture insert (Millipore) pre-coated with poly-L-lysine (100 µg.mL^−1^) and laminin (10 µg.mL^−1^). Slices were cultured for 48 hours in slice culture medium^52^ at 35 °C and 5% CO_2_ after which they were fixed with 4% PFA overnight at 4 °C, rinsed in PBS and mounted on microscope slides using Fluoromount-G mounting medium (SouthernBiotech).

### Proliferation assay

Embryonic neocortices E14.5 were dissociated as previously described^53^ and seeded (4.10^4^ cells.mL^−1^) on glass coverslips pre-coated with polyornithine (0.5 mg.mL^−1^; Sigma) and laminin (20 µg.mL^−1^; Gibco) in serum-free medium (Neurobasal; 1% B27, KCl 25 mM, 1X Glutamax, Glucose 3 g.L^−1^, penicillin/streptomycin). Eph and ephrin recombinant protein containing a human Fc fragment or the recombinant human Fc fragment only for control (8 µg.mL^−1^; R&D) were clustered for 1hr at 37 °C with anti-human Fc antibody (20 µg.mL^−1^). Pre-clustered recombinant proteins were added to the culture medium with BrdU 0.2 µM. After 24 hours at 37 °C with 5% CO_2_, cultures were fixed with 4% PFA, stained with mouse anti-TuJ1 (1:1,000; MM3-435P, Covance), incubated with HCl 2 M for 15 min at 37 °C, rinsed with Borate buffer and labeled with rat anti-BrdU (1:20; OBT0030S, AbD Serotec) and nuclei were counterstained with Hoechst. Coverslips were mounted on microscope slides using Fluoromount-G mounting medium (SouthernBiotech). The coverslips were imaged (20 random fields/coverslip; operator blinded to the experiment) using a Zeiss Imager. Z1 microscope with an Axiocam HRm digital camera and Axiovision 4.8.2 software (Zeiss). The images were blinded and the number of BrdU+ cells counted, after applying a grid, using the CellCounter plugin in the software Fiji. The resulting values were unblinded and imported into GraphPad Prism 6.0 for Mac OSX for statistical analysis and graphing. A one-way non-parametric Kruskal-Wallis test was used to test for significance.

### Tissue preparation for immunolabeling

Animals received a lethal dose of sodium pentobarbital (100 mg.kg^−1^) and were transcardially perfused with 0.1 M heparinised sodium phosphate buffer (PBS) containing 0.1% sodium nitrite to optimise vasodilation, followed by 4% paraformaldehyde (PFA) in PBS. Mouse embryos harvested from time-mated females were anesthetized on, quickly decapitated, and the brains dissected. All brains were postfixed in 4% PFA for 24 h at 4 °C. Electroporated embryonic brains were embedded in 3% LMP and 100 µm sections cut using a Vibratome (Leica). Following post-fixation, wild type (non-electroporated) embryonic brains and all postnatal brains were cryoprotected in PBS- 20% sucrose, embedded in optimal cutting temperature (OCT) medium, frozen in −45 °C isopentane and stored at −80 °C. Coronal cryostat sections of embryonic brains (18 µm) were collected on Superfrost slides and stored at −20 °C. Free floating sections (40 µm; coronal plane) were collected in serial order and stored at −20 °C in a cryoprotectant solution (50% 0.05 M phosphate buffer, 30% ethylene glycol, 20% glycerol).

### Immunolabeling

Each staining was repeated at least 3 times independently. The representative images included represent results that were consistently observed. Free-floating sections were washed in PBS, while slide-mounted sections were rehydrated in PBS, before being incubated for 1 hour at room temperature with the blocking solution (10% Normal Goat Serum, 0.3% Triton X-100 in PBS). Due to low sensitivity, sections labeled with rabbit Anti-ALDH1L1 were incubated for 2 min in citrate buffer at 90 °C for antigen retrieval. The following primary antibodies were incubated overnight at 4 °C in the blocking solution: rabbit anti-ALDH1L1 (1:500; ab87117, Abcam), rat anti-BrdU (1:20; OBT0030S, AbD Serotec), mouse anti-calbindin (1:1,000; CB38, Swant), goat anti-Doublecortin (1:200; ab18723, Abcam), rabbit anti-ephrin-A2 (1:300; SC-912, Santa Cruz), mouse anti-GAD65 (1:500; ab26113, Abcam), mouse anti-GAD67 (1:500; MAB5406, Merck-Millipore), chicken anti-GFP (1: 1,000; ab13970, Abcam), rabbit anti-Iba1 (1:500; 019–19741, Wako), rabbit anti-Ki67 (1:1,000; ab16667, Abcam), rabbit anti-Nestin (1:200, ab27952, Abcam), mouse anti-NeuN (1:500; MAB377, Merck-Millipore), rabbit anti-NeuN (1:1,000; ABN78, Merck/Millipore), mouse anti-NNF (1:1,000; SMI32R, Covance), mouse anti-Olig2 (1:500; MABN50, Merck/Millipore), mouse anti-Parvalbumin (1:1,000; PV235, Swant), rabbit anti-Pax6 (1:200; 901301, Covance), mouse anti-Satb2 (1:200; ab51502, Abcam), mouse anti-Sox2 (1:200; 3579, Cell Signaling), rabbit anti-Tbr1 (1:200; ab31940, Abcam), mouse anti-TuJ1 (1:1,000; MM3–435P, Covance), chicken anti-Vimentin (1:200; AB_528505, DSHB). Sections were washed in 0.1% Tween-20 in PBS before incubation with the appropriate fluorescently conjugated antibodies Alexa Fluor 594 and 488 (1:1,000; Molecular Probes). Fluorescently labeled sections were subsequently incubated with Hoechst solution (Dako) to stain cell nuclei, washed in PBS, free floating sections were mounted onto Superfrost slides and coverslipped with Fluoromount-G mounting medium (SouthernBiotech).

### *In situ* hybridisation

Digoxygenin-labelled riboprobe for murine *efnA2* (NM_007909.3; nucleotides 158–1002) cloned into pGEMT was used as previously described^54^.

### efnA2 KO cell quantification

The experimenter was blinded to the genotype of the animals until after all the counting were completed. Low-magnification photomicrograph (1300 × 1030 dpi) of calbindin and NeuN stained sections of adult wild type and *efnA2* ^−/−^ brains were acquired using a Zeiss Imager. Z1 microscope with an Axiocam HRm digital camera and Axiovision 4.8.2 software (Zeiss). Labeled cells were counted using the Fiji CellCounter plugin after applying a 10,000 µm^2^ grid. 4 squares spanning the neocortical thickness were counted, 3 rows/images, 160 µm interval between sections, n = 3 for NeuN counting and n = 4 for calbindin. Statistical analysis and graphing of the data was performed using GraphPad Prism 6.0 for Mac OSX. The one-way non-parametric Mann-Whitney U test was used to compare two experimental conditions, for which the normal distribution could not be assumed.

### In utero electroporation cell quantification

Coronal sections from electroporated brains collected at E18.5 (3 dpe) were incubated in Hoechst, mounted on glass microscope slides and coverslipped using Fluoromount mounting medium. Electroporated animals collected at P12 (15 dpe) were processed for immunolabeling, including GFP, as described above. For quantification of the number of GFP+ cells per compartment, low magnification images were captured to include the electroporated region from inner to outer surface, using a Zeiss Imager. Z1 microscope with an Axiocam HRm digital camera and Axiovision 4.8.2 software (Zeiss). Images were blinded using a script available in the free image analysis software Fiji. The entire electroporated region was counted, using a grid to facilitate the process. An average of 7.5 images/animal (between 5 and 10) was analyzed for E15.5+ 3 dpe experiments; 4 to 5 images were analyzed for E15.5+ 15 dpe. For the quantification of proliferative cells at E15.5+ 3 dpe, sections were imaged at higher magnification, Z-stacks were captured using an ApoTome and converted to maximum intensity projection. The resulting images were then processed as described above; with an average of 4 images/animal analyzed. The data were unblinded prior to statistical analysis and graphing using GraphPad Prism 6.0 for Mac OSX. The one-way non-parametric Mann-Whitney and Kruskal-Wallis tests were used to compare two experimental conditions and more than two experimental conditions, respectively.

### Slice assay cell quantification

Sections exhibiting extensive pyknosis, suggestive of cell death, revealed by Hoechst staining and sections where the electroporation was not restricted to the POA were systematically excluded from the analysis. Z-stacks of the electroporated regions were acquired with a Leica SP5 multichannel confocal microscope running the software LAS AF Lite. Fiji was used to generate maximum intensity projections. Using the freehand tracing tool in Illustrator (CS3), the outline of the electroporated region of the ventricular zone was drawn, defining the origin of migration (0 µm) and parallel lines were pasted at 100 µm interval. The images were saved in a tiff format. The number of GFP+ cells in each bin was counted using the CellCounter plugin in Fiji after applying a grid. The resulting counts were imported into Excel, unblinded and imported into GraphPad Prism 6.0 for Mac OSX for statistical analysis (unpaired Student’s t-test) and graphing.

### Data availability

All relevant data are available from the authors.

## Electronic supplementary material

Supplementary Data
