# Supplementary material for: Ephrin-A2 regulates excitatory neuron differentiation and interneuron migration in the developing neocortex
Source: Sci Rep. 2017 Sep 18;7:11813. doi: 10.1038/s41598-017-12185-x (PMC5603509; doi:10.1038/s41598-017-12185-x)
Supplement: Supplementary file 1 — Supplementary Data [file 41598_2017_12185_MOESM1_ESM.doc]

**Ephrin-A2 regulates excitatory neuron differentiation and interneuron migration in the developing neocortex**

Jihane Homman-Ludiye^1^, William C. Kwan^1^, Mitchell J. de Souza^1^, Jennifer Rodger^2^, James A. Bourne^1*^

^1^Australian Regenerative Medicine Institute, Monash University, Clayton, VIC 3800, Australia

^2^School of Animal Biology, the University of Western Australia, Crawley, WA 6009, Australia

**Supplementary figure 1** The neuropil of pyramidal neurons is reduced in the patches of low NeuN+ cell density although histological stain reveals normal cell architecture

***a*** *efnA2* KO neocortex coronal section labelled with NeuN to reveal patches of low neuronal density and ***a’*** normal architecture on the contralateral hemisphere. Adjacent section labelled with the cytoskeletal marker nonphosphorylated neurofilament (NNF) reveals reduction of the dendritic tree in the affected zone (***b***, boxed area) and normal architecture in the unaffected opposite hemisphere (***b’***). *efnA2* KO neocortex coronal section labelled with the nuclear stain Hoechst (***c***) and NeuN to reveal patches of low neuronal density (***c’***). The same section was then counterstained with haematoxylin and eosin (H&E) to reveal normal nuclear and cytoplasmic structure in the NeuN depleted patch (***d***, ***d’***; boxed region). Scale bar ***b’***; ***c***-***d’*** 200 µm


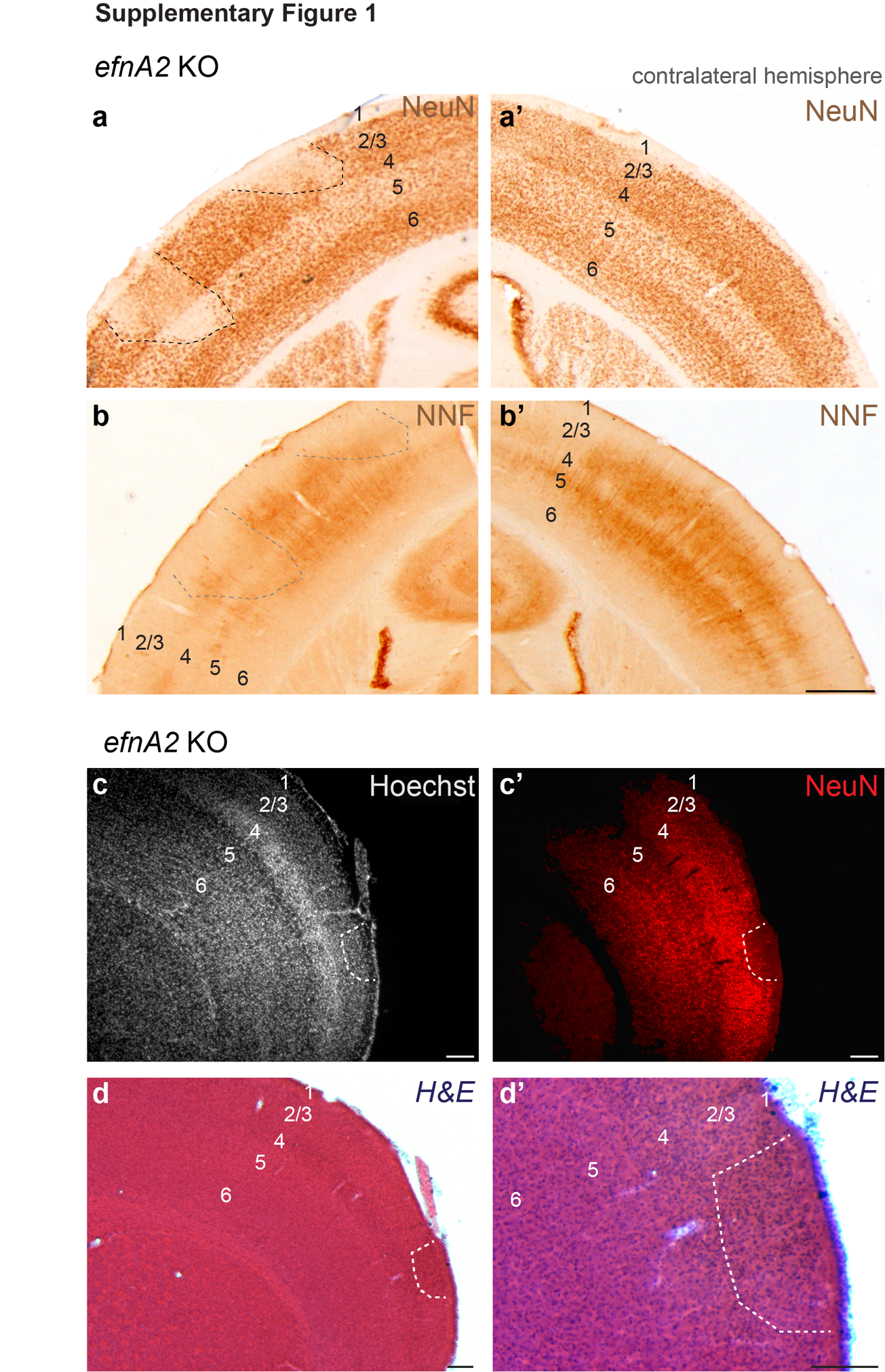


**Supplementary Table 1** Expression level of cell type specific markers in the low-NeuN density patches in *efnA2* KO neocortex

| **Progenitor cells** | **Neurons** | **Interneurons** | **Macroglia** | **Microglia** | **Expression level compared to control** |
| --- | --- | --- | --- | --- | --- |
| Doublecortin, Nestin, Sox2, | ⤫ | ⤫ | ⤫ | ⤫ | *Not expressed* |
| Vimentin | NNF, Tbr1, Satb2 | Calbindin, Parvalbumin | Olig2, ALDH1L1 | ⤫ | *Reduced* |
| ⤫ | ⤫ | ⤫ | ⤫ | Iba1 | *Unchanged* |
